# A randomized controlled comparison of pembrolizumab and chemotherapy in patients with ipilimumab-refractory melanoma

**DOI:** 10.1186/1479-5876-13-S1-O5

**Published:** 2015-01-15

**Authors:** Reinhard Dummer, Adil Daud, Igor Puzanov, Omid Hamid, Dirk Schadendorf, Caroline Robert, Jacob Schachter, Anna Pavlick, Rene Gonzalez, F Stephen Hodi, Lee D Cranmer, Christian Blank, Steven J O’Day, Paolo A Ascierto, April K S Salama, Nicole Xiaoyun Li, Wei Zhou, Joy Lis, Scot Ebbinghaus, Peter S Kang, Antoni Ribas

**Affiliations:** 1 Department of Dermatology, University of Zurich, Zurich, Switzerland; 2 Helen Diller Family Comprehensive Cancer Center, University of California at San Francisco, San Francisco, CA, USA; 3 Division of Hematology-Oncology, Vanderbilt-Ingram Cancer Center, Nashville, TN, USA; 4 Melanoma Center, The Angeles Clinic and Research Institute, Los Angeles, CA, USA; 5 Department of Dermatology, University Hospital Essen, Essen, Germany; 6 Dermatology Unit, Department of Medicine, Institut Gustave Roussy, Villejuif, France; 7 Oncology Division, Sheba Medical Center, Tel Hashomer, Israel; 8 Department of Medicine, New York University Perlmutter Cancer Center, New York, NY, USA; 9 Division of Medical Oncology, University of Colorado Denver, Denver, CO, USA; 10 Melanoma Center, Dana-Farber Cancer Institute, Boston, MA, USA; 11 Department of Hematology/Oncology, University of Arizona Cancer Center, Tucson, AZ, USA; 12 Department of Medical Oncology, Netherlands Cancer Institute, Amsterdam, Netherlands; 13 Department of Medical Oncology, Beverly Hills Cancer Center, Beverly Hills, CA, USA; 14 Unit of Medical Oncology and Innovative Therapy, National Tumor Institute Fondazione “G. Pascale,” Naples, Italy; 15 Department of Medicine, Duke Cancer Institute, Durham, NC, USA; 16 Merck & Co., Inc., Whitehouse Station, NJ, USA; 17 Jonnson Comprehensive Cancer Center, University of California at Los Angeles, Los Angeles, CA, USA

## Background

Pembrolizumab blocks the interaction between PD-1 and its ligands PD-L1 and PD-L2, thereby inducing an antitumor immune response. In a phase I study, pembrolizumab demonstrated promising antitumor activity and acceptable safety in patients with ipilimumab-treated melanoma, leading to accelerated approval in the US.

## Materials and methods

KEYNOTE-002 is a randomized phase 2 study in patients with ipilimumab-refractory melanoma (ie, confirmed PD in the 24 weeks following ≥2 ipilimumab doses) and, if *BRAF* mutant, previously treated with a BRAF inhibitor. Patients were randomized 1:1:1 to pembrolizumab 2 or 10 mg/kg Q3W or investigator-choice chemotherapy (carboplatin + paclitaxel, carboplatin, paclitaxel, dacarbazine, or temozolomide). Patients with PD confirmed by independent central review could cross over to pembrolizumab treatment after the first 3-month assessment. Primary objective of the interim analysis prespecified to occur after ≥ 270 PFS events (RECIST v1.1, independent central review) was to evaluate the superiority of either pembrolizumab dose over control for PFS at a 1-sided 0.25% significance level (estimated HR 0.66).

## Results

From Nov 2012 to Nov 2013, 540 patients from 12 countries enrolled. Based on central review of a total of 410 PFS events, the HR was 0.57 and 0.50 for pembrolizumab 2 and 10 mg/kg Q3W, respectively, over control (*P*<0.00001 for both comparisons). The 6-month PFS rate was 34% (95% CI 27%-41%) and 38% (95% CI 31%-45%) for pembrolizumab 2 and 10 mg/kg, respectively, compared with 16% (95% CI 10%-22%) for the control arm. PFS by investigator assessment was similar to that of central review. The PFS effect was consistent in all subgroups. ORR was 21% at 2 mg/kg, 25% at 10 mg/kg, and 4% in the control arm (*P*<0.0001 for both comparisons). Median response duration was not reached in either pembrolizumab arm and was 37 weeks in the control arm. Forty-eight percent of patients in the control arm crossed over to pembrolizumab treatment. OS data are not mature (final OS analysis will be performed after 370 deaths have occurred). The safety profile was consistent with that previously observed for pembrolizumab. Despite a decreased therapy duration, rates of grade 3-5 drug-related AEs were numerically higher in the chemotherapy control arm (26%) than in the pembrolizumab 2-mg/kg (11%) and 10-mg/kg (14%) arms.

## Conclusion

Both pembrolizumab doses met prespecified criteria for PFS superiority over the chemotherapy control arm. Pembrolizumab significantly prolongs PFS compared with chemotherapy, approximately doubling the 6-month rate in an ipilimumab-refractory population.

## Clinical trial registration number

NCT01704287

